# Hospital and Institutional News

**Published:** 1914-05-16

**Authors:** 


### HOSPITAL AND INSTITUTIONAL NEWS.

### A HOSPITAL PRESIDENT AS GOVERNOR-GENERAL.

The appointment of Prince Alexander of Teck to succeed the Duke of Connaught as Governor-General of the Dominion of Canada, while a striking example of the hard work now expected from members of our Royal Family, will mean a personal regret to the authorities of the Middlesex Hospital, whose president he has been for several years. It is hardly necessary to remind our readers that the association of this hospital with this branch of the Royal Family is peculiarly close, from the time when his brother, the late Prince Francis, undertook the presidency in the midst of the financial difficulties out of which he helped to pilot the Middlesex Hospital by his active work on behalf of the appeal associated with his name. We all remember that his untimely death prevented Prince Francis from seeing the fruition of his work, but we remember also the prompt manner in which Prince Alexander took his post, and spent his energies in carrying through the task which his brother had begun so successfully.

### SEPTIC THROATS IN THE STAFF AT CAMBRIDGE.

The number of out-patients at Addenbrooke's Hospital, Cambridge, has increased so much of late, "notwithstanding," in the words of the Rev. J. B. Lock, who presided at the quarterly meeting last week, "the Insurance Act," that there is more need than ever for the new out-patient department, which, among improvements in process of realisation, was noted in our Report in The Hospital of January 31 last. The new wards are nearing completion, and the value of the work performed is happily shown by a donation of £100 from Baron von Hugel, the eminent author of "The Mystical Element of Religion," which he has ear-marked for the building fund. The donor mentioned that his gift was "in recognition of the great kindness shown to the sick Catholics who have been admitted." There was, however, one disquieting fact announced at the meeting last week—namely, the fact that two resident medical officers and several members of the nursing and domestic staffs have been off duty with septic throats. No public mention seems to have been made of the cause, which, in such a well-managed institution, must, no doubt, have been carefully investigated. What was it?

### THE BUDGET AND PATHOLOGICAL RESEARCH.

An announcement appears in the Budget that a certain sum will be set aside for pathology for the purpose of aiding Municipalities in the diagnosis of disease. It will be interesting to record the progress that has already been made in this direction. A circular has been issued by the Local Government Board drawing the attention of the various Boroughs throughout the Kingdom to the necessity of providing facilities where tubercle bacilli, widal, etc., can be dealt with. As the Chancellor of the Exchequer pointed out, many of the Boroughs have shown considerable apathy in regard to this matter, but, on the other hand, there are Boroughs alive to their obligations who have seized the first opportunity to place at the disposal of the medical graduates within their area such facilities as may tend towards the improved health of the community. To this end the Metropolitan Boroughs of Greenwich, Woolwich, and Deptford have been some of the first to adopt a system of pathological examinations. These three Boroughs have placed their pathological work in the Laboratories of the London School of Clinical Medicine which is attached to the *Dreadnought* Seamen's Hospital at Greenwich, and now the Borough of West Ham has taken a like course and entered into an arrangement for its pathological work to be performed in the London School of Tropical Medicine which is attached to the Albert Dock Hospital of the Seamen's Hospital Society.

### THE GOVERNMENT AND PATHOLOGICAL LABORATORIES.

It is well known that it is the desire of the Government that pathological laboratories where they exist shall be made use of rather than that separate establishments be set up. First, on account of the expense; and, secondly, because it will not be found feasible to provide as efficient and large a staff for these Borough laboratories as exist in the general hospitals. Furthermore, there is the question of Sunday and night work. A hospital never sleeps, and there are always facilities for dealing with urgent cases, such as diphtheria. Besides this, in a hospital laboratory no specimen, no matter how trivial, is reported upon by a non-professional assistant. Every specimen comes under the observation of a medical graduate. It is remarkable how the work has grown within the last few years. The aid to the busy practitioner is incalculable and the satisfaction of confirmed diagnosis enables the busy man to get through more work with greater confidence, as well as with greater advantage to those who come under his care.

### PROGRAMME OF THE NEWCASTLE CONFERENCE.

The fifth annual conference of the British Hospitals Association, of which preliminary particulars have been published in The Hospital, will take place at Newcastle-upon-Tyne, from June 18 to 20. The details of the programme have now been settled. By kind permission of the governors the meetings will be held in the Library of the Royal Victoria Infirmary, situated in Queen Victoria Road, and the Lord Mayor of Newcastle, Councillor Johnstone Wallace, J.P., will act as president of the conference. The deputy-chairman will be Sir George Henry Philipson, M.D., chairman of the Royal Victoria Infirmary. Proceedings will begin on Thursday, June 18, at 10 a.m., with an address of welcome by the Lord Mayor, whom Mr. G. H. Hume, M.D., F.R.C.S., vice-president, will follow with a paper entitled "The Voluntary Hospital on its Trial," to be succeeded by a general discussion. After lunch at 1 P.M. the wards and various departments of the Royal Victoria Hospital will be visited, and at 3.30 the Lord Mayor will hold a reception and garden party in Leazes Park and in the contiguous grounds of the Royal Infirmary.

### NEWCASTLE CONFERENCE: SECOND DAY.

On the morning of Friday, June 19, the Sheriff of Newcastle, Councillor Herbert Shaw, will read the first of three papers on "The Voluntary Hospitals and their Relation to Social Service Work." The subject of the second paper will be "The Proposed Bill for the Amendment of the Truck Acts: How Sub-Section *(e)* of Section 1 might Affect the Voluntary Hospitals," by Mr. William Straker, corresponding secretary, Northumberland Miners' Association, member of the house committee of the Royal Victoria Infirmary. A very interesting discussion is expected. The third paper, by Mr. P. J. Michelli, C.M.G., secretary and superintendent of the Seamen's Hospital, Greenwich, will be upon the subject of "Pensions for Hospital Officers." After questions and discussion have exhausted themselves on this point a business meeting will be held to elect a new council and honorary officers for the year. The afternoon will probably be devoted to an excursion. Those who are enabled to stay over Saturday will have the opportunity of making a steam-trip on the River Tyne, and for visits to Durham Cathedral, Castle, University, and the Roman Wall. We strongly urge as many members as possible to stay if possible over Saturday in order to make these trips, and, indeed, it would be a good policy on the part of every hospital committee to insist upon their representatives doing so, for it is when closeted in a tram-car or train, or snugly cooped up in a river steamer, that confidences are exchanged and difficulties discussed, and all important points of detail and experience elaborated.

### PERSONALIA AT THE CONFERENCE.

Councillor Johnstone Wallace, J.P., the Lord Mayor, is generally regarded not merely as one of the most influential, but also one of the ablest men in Newcastle, and his address of welcome will probably pass beyond the formality which is its occasion, and review the situation from the point of view of Northern hospitals as a whole. The proposed amendment of the Truck Acts, for example, is a subject of far greater primary interest to Northern than Southern institutions, because of the obvious fact that it is in our manufacturing centres in the North that the working-class subscriptions to hospitals have assumed an importance analogous to that of the great Funds in London. Councillor Herbert Shaw, the Sheriff, who reads the first paper on Friday, is well known locally as a public orator and platform speaker, and few local "demonstrations" are complete without his support or opposition on one side or the other. Mr. William Straker now represents about 50,000 miners and has worked his way to an influential position by pertinacity, hard work, and administrative capacity. Last, but not least, Mr. Hume, who is consulting physician to the Royal Victoria Infirmary, is also a hospital historian and author of the History of the Royal Victoria Infirmary, Newcastle. The Hospital, by the way, was able to point out an omission in Mr. Hume's admirably written and comprehensive work a little more than a year ago, when in an article published on page 534 of our issue of February 15, 1913, we drew attention to the fact that the wooden tablet affixed to the walls of the Victoria Infirmary's out-patient department, relating to the Pigg Charity, corroborates a quotation in Tyneside dialect from Surtees' "Handley Cross." Who and what John Pigg was, and whether his descendant really had a grievance against him for "willing away arle wor brass to the Formory" was discussed in the article which forms a curious addendum to Mr. Hume's fine work.

### A WELL-KNOWN SECRETARY'S RESIGNATION.

At a special meeting of the general committee of the Oxford Eye Hospital the resignation was announced of Sir A. Frederick Bradshaw, K.C.B., from the post of honorary secretary, and also from that of trustee. Sir Frederick, who has held the post of honorary secretary for thirteen years, was born in 1834, and entered the Army Medical Department in 1857. A "Bart.'s" man he qualified in 1856, took the M.R.C.S. in 1857 and the M.R.C.P. in 1882. He was in India during the Mutiny, and was present at the siege of Lucknow, and from 1863-64 he held the post of garrison and brigade surgeon at Delhi. Later, from 1884-86, he became principal medical officer of the Quetta District, Baluchistan, and held similar posts at home as well as in India and Egypt, and received the C.B. in 1891. From 1892-95 he became principal medical officer of His Majesty's Forces in India, and retired in 1895 after thirty-five years' service in that country. In seconding a vote of thanks to Sir Frederick for his services, Mr. Doyne recalled the fact that he had been one of his proposers or seconders on his appointment, and that he had made a good diagnosis when he had asserted that Sir Frederick, though then sixty-seven years of age, was the youngest man in the room.

### A CHAIRMAN ON A SECRETARY'S WORK.

Mr. Mentieth Ogilvie, chairman of the house committee, in seconding the vote of thanks, remarked that very few people knew how heavy was the work of a hospital secretary, though many had learnt to know "the wonderful faculty for writing persuasive letters" which Sir Frederick possessed. After the motion of thanks had been carried unanimously Col. Arthur W. Forbes was elected honorary secretary of the general committee, and Mr. F. Mentieth Ogilvie was elected a trustee. The Rev. W. E. Sherwood, the Mayor, presided, and the Rev. G. B. Cronshaw, treasurer of the

Radcliffe Infirmary, who acted with such charming hospitality towards the British Hospitals Association Conference at Oxford last year, was also present.

### THE WARD LOCKER COMPETITION: WHO ARE THE CULPRITS?

We are gratified to note that a number of the principal officers of important hospitals have entered this competition, and are so kindly co-operating to make these competitions, and the scheme of which the present is the forerunner, as great and practical a success as possible. We hope that every secretary and matron, and some of the older sisters at any rate, will kindly refer to The Hospital of last week and vote in this competition, because from their practical knowledge and wide experience their opinion is of great importance. All voting papers must reach 28 Southampton Street, Strand, W.C., not later than Saturday, May 16, 1914. We regret to notice that someone has evidently organised an attempt by combination to obtain the Special Prize. It may be well, therefore, to intimate at this stage that we have taken full precautions against proceedings of this reprehensible character, and that those who indulge in them will be disqualified and their voting papers cancelled. The aim of The Hospital in this department, as in all other departments of the paper, is to prove of as great practical help as possible to every intelligent worker throughout the hospital field. We rely upon the high standard of personal honour and the spirit which unites all zealous workers in the cause of the sick to co-operate with us in preventing the success of any attempt to mis-use the Special Prize Scheme. We beg to intimate that the Special Prize will only be given to the voter whose coupon is found most nearly to agree with the majority of technically accurate opinions based upon competent knowledge as proved by the voting. This intimation supersedes that published last week on page 164 in connection with this Prize Scheme.

### SPECIALIST STAFFS FOR POOR-LAW INFIRMARIES

The Ashton-under-Lyne Guardians have had under consideration the question of the reorganisation of the medical staff of their workhouse infirmary. The matter was referred to a committee, who had before them a report from Dr. Hughes, the present medical officer, recommending the appointment of two resident medical officers, a visiting surgeon, and a visiting ophthalmic surgeon. The committee finally decided to recommend the appointment of a resident medical superintendent at a salary of £350, rising to £400, with rations, etc.; of an assistant medical officer; and of an ophthalmic surgeon at an honorarium of £25 per annum. The infirmary possesses 300 beds, so that the suggested staff cannot be considered a generous one. It is, however, interesting as showing that Guardians are beginning to realise the need of appointing a specialist staff—a need to which we have frequently drawn attention. An ophthalmic surgeon is one of the most obvious requirements of every Poor-Law infirmary. Yet there are only about half-a-dozen Boards of Guardians in the whole of England and Wales who have realised this and have appointed such an officer.

### THE DEADLOCK AT DROITWICH.

In our issue of April 4 we referred to the fact that the Droitwich Guardians had been unable to obtain a district medical officer for a salary of £90 per annum. The Board consequently increased the salary to £100, but this, also, failed to attract any candidate. The local medical men have unanimously decided not to apply for the post until the salary has been further increased. They point out that the late medical officer was receiving £120, and about £30 in extra fees for vaccination and midwifery, and that these fees will be much smaller in the future. The Guardians finally decided to appeal to the Local Government Board asking for information and instruction as to what was to be done. It is difficult to see what the Guardians expect to gain by this appeal. Medical men cannot be forced to accept a post which they regard as inadequately remunerated, and the only course open to the Guardians is to confer with the local practitioners and to arrive at an agreement with them.

### THE ASYLUM WORKERS' ASSOCIATION.

On Wednesday next, May 20, the annual meeting of the Asylum Workers' Association will be held at 11 Chandos Street, Cavendish Square, at 3 p.m. Sir John Jardine, K.C.I.E., M.P., the president of the Association, will take the chair, and after the report has been submitted, and the officers elected, presentations will be made of medals for long and meritorious service. Dr. G. E. Shuttleworth, the honorary secretary, will supply all particulars on application.

### PHYSICIAN'S CLAIM TO PEERAGE ALLOWED.

In 1911 three claimants came forward to urge their right as co-heirs to the baronies of Burgh, Strabolgi, and Cobham, which were in abeyance, and petitioned the King to determine the abeyance in their favour. One of them was Dr. Reginald Gervase Alexander, of Blackwall Lodge, Halifax, consulting physician to the Bradford Royal Infirmary and to the Halifax Royal Infirmary, at both of which formerly he had held the post of senior physician. The hearing of this claim was concluded before the Committee of Privileges of the House of Lords last week. The result was that the Committee were unanimous that, in Lord Halsbury's words, the claimants had made out their case. Dr. Alexander graduated at Cambridge in 1874, and took the M.D. degree of Edinburgh seven years later. He has published "The Prolongation of Life" and "Phthisis: Its Prevention and Cure." Recently left a widower, he has two sons and two daughters. The eldest son is himself a medical practitioner; and the second, who formerly studied as a solicitor, and held a post under the Public Trustee, on one occasion contributed a valuable article to The Hospital, in a series written, as its general title implied, by laymen. In dealing with Dr. Alexander's claim in The Hospital of July 6, 1912 (page 341), we paid a tribute to the equally masterly literary style and technical knowledge on points of Privilege possessed by Mr. J. Horace Round. Mr. Round has since become, we believe, official adviser to the Crown in Privilege cases.

### DO COTTAGE HOSPITALS GET ALL THE LEGACIES?

In complaining that the Royal Surrey County Hospital, Guildford, had received no legacy during the past year, General Sir Edmond Elles, G.C.B., G.C.I.E., the hon. treasurer of the institution, said that the reason for this absence of support was, he thought, to be found in the present-day tendency to leave legacies to cottage hospitals. Cottage hospitals, he added, come more before the public. This interesting point of view is one which the secretaries of larger hospitals may well discuss, for its implication is that, in the truest sense of the word, the advertisement of the cottage hospital (meaning efficiency as well as publicity) is better managed, or establishes more personal relations with the public than the big hospital is able to do. General Elles notwithstanding, we find few legacies in cottage hospital accounts. Is this the case? What are our readers' opinions?

### DISTRICT MEDICAL OFFICERS AND OPERATION FEES.

When the Departmental Committee which is at work upon the revision of the Poor-Law Orders has had time to consider the duties of the district medical officer it will, no doubt, considerably expand the table of operations for the performance of which the district medical officer is entitled to a special fee. The present table was drawn up in 1847, and the only operations included in it are amputations and operations for strangulated hernia. At that time surgery was in its infancy, and these were the only operations likely to be urgently required. In all cases that were not urgent the Poor-Law Commissioners advised that a Poor-Law patient should be sent to a hospital for any surgical operation deemed necessary. Article 181 of the Consolidated Order of 1847, however, empowers the Guardians in any surgical case, not specifically provided for, which has presented peculiar difficulty or required and received long attendance from the district medical officer to make to him such reasonable extra allowance as they may think fit. These powers have not been acted upon very generally, and district medical officers legitimately complain that they are often asked by their Guardians to perform without any additional remuneration operations which ought not to be considered part of their ordinary work. Of recent years, since the introduction of the medical examination of school children, cases of enlarged tonsils and adenoids are often referred to the district medical officer in rural districts for operation, without any extra remuneration being paid. This is obviously unjust, especially in the case of the older district medical officers, whose appointments often date from before the time when these operations were in general use. There are also many urgent operations performed nowadays which were not dreamed of in 1847. Such operations include laparotomy for appendicitis or ruptured gastric ulcer, operations for empyema, mastoid abscess, and so on. It is no doubt better, whenever this condition permits removal, that a poor patient should be sent to a Poor-Law infirmary or voluntary hospital for operation; but when, for any reason, the operation is performed by the district medical officer he should receive a special fee for the work.

### THE SALARIES OF SANATORIUM SUPERINTENDENTS.

At a meeting of the Nuneaton Joint Sanatorium Committee, at which accounts were presented showing the receipts and expenditure for the five months since the institution was opened as a sanatorium, a letter was read from Dr. Joseph, the medical superintendent, asking that his salary should be increased from £105 to £200. It was stated that there are now twenty-eight patients under treatment, and Mr. W. J. Phillips urged that some attempt at after-care work should be undertaken, so that something should be done to keep in touch with the patients on their discharge. It was eventually decided after discussion to raise the medical superintendent's salary to £150. In dealing with this question we have urged again and again the importance of every new institution attracting the skilled tuberculosis administrator at the start by the offer of a salary good enough to attract really able and enthusiastic men. One reason why this is so important is that an authority which economises on salaries at the start only makes matters worse, should it have chosen the wrong man as so often happens, by raising his salary as is often done a year or two later.

### THE "UNHONOURED" STATUS OF GUARDIANS.

Mr. G. E. Lloyd-Baker presided over the West Midland Poor-Law Conference at Great Malvern last week, at which Mr. H. Allen Armitage read a paper explaining the provisions of the new Poor-Law Orders. He said that progressive Boards had carried out the spirit of the new Orders before they were issued, but, owing to the lack of public interest in Poor-Law work, the status of the Guardian was not honoured as much as its duties required, and progressive policy had not been supported. If classification were to be made to apply to the small Unions it must be by the combination of Boards for dealing with the several classes of the poor. Furthermore, he urged that in this classification the needs of the institution officers must not be lost sight of. In many institutions, he added, the accommodation for the officers was totally inadequate. The confinement of institutional life was hardly understood by those outside it, and officers were, after all, very much what Guardians made them.

### AN ALTERNATIVE TO COMBINED UNIONS.

In the following discussion, Mr. Simpson, of Bristol, urged that too low an age had been fixed for children not to be in the workhouse. Mr. Everest, of Atcham, said that if Guardians did not want to be abolished they must move, and the only move which they could make in rural districts was to combine. Mrs. Walter Brown, of Worcester, enlarged on the danger of classification, and asserted that the old people would not like being left to themselves without the society of the young. Mr. R. Waite, of Birmingham, pointed out that the difficulties of classification did not cease with combination, and that the pigeon-hole principle should not be pushed to an extreme. The discussion was wound up in a very instructive manner by one of the Local Government Board inspectors of the district, Mr. Wethered, who said that, where rural Unions could not combine, scattered homes, for boys and girls respectively, was the best plan to adopt. If people were removed from their own districts the existing reluctance to enter the workhouse would be increased. The relation of the status of the Poor-Law Guardian to the slow progress so characteristic of certain Unions was a point which Mr. Allen Armitage may be congratulated for bringing out.

### OUR REPORT AND ITS SEQUEL AT GUILDFORD.

Mr. F. F. Smallpiece, chairman of the Royal Surrey County Hospital at Guildford, referred at the annual meeting last week (of which, by the way, a model report appeared in the *West Sussex Gazette*) to Sir Henry Burdett's recommendations. His Report on this institution appeared in our issue of February 28 last, some months after his visit, and it laid stress on the need for "arrangements to provide the house surgeon with his own sitting-room, and also to give similar accommodation to the two junior resident medical officers." Mr. Smallpiece said that the structural improvements suggested had received the careful attention of the committee, and that the smaller matters were being dealt with. He added that the suggested improvement of the sterilising department will probably cost £300; and as to the better accommodation of the resident medical staff, "I do not think," Mr. Smallpiece added, "there will be any difficulty, except on the ground of expense." As, however, the jubilee appeal for an additional annual income of £1,200 has already produced an increase of rather more than £800, we are encouraged to believe once more that efficiency is the best of all hospital advertisements, and that the way to keep efficient is to interest the locality in every improvement which the hospital requires.

### A WELL-EDITED ANNUAL REPORT.

We congratulate the board of the Royal Buckinghamshire Hospital, Aylesbury, and the secretary upon the great improvement in the appearance and the arrangement of the annual report for 1913 just issued. It is in marked contrast to the former reports of a few years ago, and shows that someone active in the management is conscious of the importance of making the annual report an attractive and readable document. The illustrations are good of their kind, and help to make the report interesting and useful. It is, however, serious evidence of a want of apprehension of public opinion in regard to voluntary hospitals, especially where, as at Aylesbury, they depend almost entirely upon the free gifts of the public, that no change has been made in the way in which the accounts are presented, and that neither the accounts nor the books are at present kept upon the Uniform System. Until the Uniform System is introduced at Aylesbury it will not be possible to compare the working and progress of this hospital in all departments with those of other institutions of its size and type. We hoped when Mr. S. E. Wilkins became secretary that he took up the work with a determination to put everything in order and quickly. The accounts at Aylesbury call aloud for his immediate attention and that of the committee.

### THE DEARTH OF HOUSE SURGEONS AND HOW TO MEET IT.

It is an ill wind that blows no one any good, and the introduction of the panel system seems to have had the effect of materially increasing the relatively small emolument hitherto paid to house surgeons up and down the country. Thus one hospital, the Beckett Hospital, at Barnsley, which is, we believe, in most respects comfortable and an attractive field of labour for a resident, is offering larger salaries—namely, £200 and £160 respectively, as our advertising columns show. We hope that this liberality on the part of the committee may meet with the success it deserves. A resident appointment is essential to all newly qualified men who are resolved to make a real success of their professional life, and to do the best work in private practice. Unlike some committees and some medical staffs the authorities at Barnsley hope the increased salaries will induce the practitioners selected for the vacant posts to remain at the Beckett Hospital for a considerably longer period than six months. We have little doubt, if they find the work congenial, interesting, and of practical value, that a longer residence may prove one outcome of the higher salaries now offered at Barnsley.

### THE REPORTING OF INQUESTS.

Only a fortnight ago in these columns we took occasion to protest against the slovenly reporting of inquests which obtains unchecked by editors in the daily papers, and which passes over the many very interesting questions which are always arising and extremely various, in favour of the purely sensational element which is mostly unvarying and quite trivial. But this slovenly reporting is a different thing from actual misrepresentation, which Mr. Ernest Augustine Gibson, the Coroner for the City of Manchester, alleged against a local newspaper last week. The case was eventually settled in his favour in court. The terms of the settlement, which received the sanction of the Court, were that the record should be withdrawn, and that the defendants should undertake to pay Mr. Gibson's costs as between solicitor and client on the principle of indemnity. The defendants also apologised. Without going into the details of the case, it appears that by publishing a very much condensed report the newspaper had the effect of making the Coroner appear in his summing-up to be dealing with one matter, when, in fact, he had been dealing with another. Letters thereupon poured in and were published fastening on the misrepresentation occasioned by the report, and public feeling became aroused sufficiently to make Mr. Gibson bring an action for libel. The case appears to have been one of bad editing, without malice of any kind, in fact an accident, but we welcome Mr. Gibson's attitude, and congratulate him on his action because we feel that anything which brings home to the editors of daily papers the fact that inquests are important public occasions, and Coroners' summings-up pronouncements which must be treated in a responsible, as opposed to a cursory and contemptuous manner, is all to the good, if the public are no longer to be deprived of the very interesting and valuable matters of public interest which are now as strictly censored by the press as if they were blue-pencilled by a Russian bureaucrat specially appointed for this purpose.

### A LADY EDUCATIONIST ON MEDICINE.

The infrequency with which observations on medical matters by outsiders can be trusted is shown rather strikingly in the current number of no less famous a periodical than the *Athenæum*. In the report of a lecture by a lady on "Biology in Relation to Education" some very uncritical dicta are met with. "The 'abdominal brain' is larger in the female than in the male . . . hence it comes that . . . boys suffer more from malnutrition than girls, and more often die young." Various marvels from history are cited with apparently full belief of the same. The seventeenth-century Spanish woman who could see through the crust of the earth or the human integument, discovering in the latter case diseases which had escaped the observation of the best physicians, every hospital resident of sociable tastes has come across a reincarnation of pretty often in the out-patient department. The Swiss lady, too, who could tell the thickness of a coal seam a mile or so under her feet by the taste it produced in her mouth, one hears of in all the best drawing-rooms. She could further discover the existence and nature of diseases, and "cure them by the touch of her hand or finger." The feminine mind, as even in Madame Montessori's case, is apt to run a little to extravagance, but the *Athenæum* is not the place in which one expects to find them occupying valuable space. What will Continental subscribers think of page 629 of its late issue? It reads for all the world like Mrs. Eddy.

### THE PHARMACEUTICAL COUNCIL ELECTION.

The Public Pharmacists' and Dispensers' Association is engaged in a vigorous campaign to secure the return of a representative on the Council of the Pharmaceutical Society of Great Britain in the person of Mr. Herbert Skinner, chief pharmacist, Great Northern Central Hospital, London. The Association points out, in the circular-letter which it has addressed to all pharmacists engaged in institutional work, that a representative could render them invaluable service and that the present seems a particularly opportune time for public pharmacists to join together by voting *solid* for their own candidate. Many reforms are now desired by sections of the pharmaceutical profession, including representation on the Local Associations Conference and one to the effect that all public dispensaries should be under the control of a registered pharmacist, as in most of the larger hospitals, London County Council mental hospitals, and H.M. Prison Service. Mr. Skinner, we understand, is pledged to attack these problems, and this active branch of institutional workers will be rewarded, it is hoped, by securing his return.

### THIS WEEK'S DRUG MARKET.

Although there is some improvement in business, the market continues on the quiet side. The first news of the earthquake in Sicily not unnaturally suggested the probability that the market for Sicilian produce such as citric acid and essence of lemon would be affected; and although it is by no means unlikely that the disaster will prove a disturbing factor in the market, later reports of the restricted nature of the shock minimise considerably the possibility of any great advance in the prices of citric acid and essence of lemon. After the disaster at the end of 1908 the price of essence of lemon was nearly trebled, but the disaster resulting from the recent shock cannot be compared with the havoc caused by the former one. In the meantime the value of citric acid is firmly maintained, with an advancing tendency, and the decline in essence of lemon has at any rate received a check. The hostilities in Mexico continue to prove a disturbing factor in the market for jalap and Mexican sarsaparilla, the prices for both of which have an upward tendency. An advance in the price of English refined camphor would not come as a surprise. Tartaric acid is in good demand. Ergot of rye is dearer. There is little change in the position of opium and morphine, but values are well maintained. Chrysarobin is dearer. The price of cod-liver oil still has an upward tendency.

### TO OUR READERS.

Contributions are specially invited from any of our readers to these columns. They should deal with topical subjects and news. They must be authenticated for the information of the Editor only. The minimum payment if published is 5s. There is no hard-and-fast rule as to space, but notes of about twenty lines in length are preferred.

